# Antigenic Challenge Influences Epigenetic Changes in Antigen-Specific T Regulatory Cells

**DOI:** 10.3389/fimmu.2021.642678

**Published:** 2021-03-23

**Authors:** Dorota Iwaszkiewicz-Grzes, Magdalena Piotrowska, Mateusz Gliwinski, Zuzanna Urban-Wójciuk, Piotr Trzonkowski

**Affiliations:** ^1^ Department of Medical Immunology, Medical University of Gdansk, Gdańsk, Poland; ^2^ International Centre for Cancer Vaccine Science, University of Gdańsk, Gdańsk, Poland

**Keywords:** TSDR, antigen-specific, DNA methylation, histone H3, gene expression, epigenetics

## Abstract

**Background:**

Human regulatory T cells (Tregs) are the fundamental component of the immune system imposing immune tolerance *via* control of effector T cells (Teffs). Ongoing attempts to improve Tregs function have led to the creation of a protocol that produces antigen-specific Tregs, when polyclonal Tregs are stimulated with monocytes loaded with antigens specific for type 1 diabetes. Nevertheless, the efficiency of the suppression exerted by the produced Tregs depended on the antigen with the best results when insulin β chain peptide 9-23 was used. Here, we examined epigenetic modifications, which could influence these functional differences.

**Methods:**

The analysis was pefromed in the sorted specific (SPEC, proliferating) and unspecific (UNSPEC, non-proliferating) subsets of Tregs and Teffs generated by the stimulation with monocytes loaded with either whole insulin (INS) or insulin β chain peptide 9-23 (B:9-23) or polyclonal cells stimulated with anti-CD3/anti-CD28 beads (POLY). A relative expression of crucial Tregs genes was determined by qRT-PCR. The Treg-specific demethylated region (TSDR) in FoxP3 gene methylation levels were assessed by Quantitative Methylation Specific PCR (qMSP). ELISA was used to measure genomic DNA methylation and histone H3 post-translational modifications (PTMs).

**Results:**

Tregs SPEC_B:9-23_ was the only subset expressing all assessed genes necessary for regulatory function with the highest level of expression among all analyzed conditions. The methylation of global DNA as well as TSDR were significantly lower in Tregs SPEC_B:9-23_ than in Tregs SPEC_INS_. When compared to Teffs, Tregs were characterized by a relatively lower level of PTMs but it varied in respective Tregs/Teffs pairs. Importantly, whenever the difference in PTM within Tregs/Teffs pair was significant, it was always low in one subset from the pair and high in the other. It was always low in Tregs SPEC_INS_ and high in Teffs SPEC_INS_, while it was high in Tregs UNSPEC_INS_ and low in Teffs UNSPEC_INS_. There were no differences in Tregs/Teffs SPEC_B:9-23_ pair and the level of modifications was low in Tregs UNSPEC_B:9-23_ and high in Teffs UNSPEC_B:9-23_. The regions of PTMs in which differences were significant overlapped only partially between particular Tregs/Teffs pairs.

**Conclusions:**

Whole insulin and insulin β chain peptide 9-23 affected epigenetic changes in CD4^+^ T cells differently, when presented by monocytes. The peptide preferably favored specific Tregs, while whole insulin activated both Tregs and Teffs.

## Introduction

T regulatory cells (Tregs) constitute a subset of CD4^+^ T lymphocytes which is pivotal in immune tolerance due to their ability to suppress effector cells. There are two main subpopulations of Tregs: natural (nTregs or tTregs) which develop in the thymus during thymopoesis and peripheral (pTregs) which differentiate from naïve CD4^+^T cells in the periphery during TCR stimulation in the presence of cytokines (e.g. IL-2, TGF-β). Natural T regulatory cells (CD4^+^CD25^high^CD127^-^FoxP3^+^; Tregs) are mainly predisposed to exert suppressive functions over effectors, which is highlighted by stable genomic architecture in this subset of Tregs. Transcriptional factor FoxP3 (forkhead box P3) is a master regulator of Tregs. Its expression in Tregs is kept stable *via* Treg-specific demethylated region (TSDR) in the promoter of *FoxP3* gene. The sustained expression of FoxP3, possible due to demethylated TSDR, allows the expression of a wide range of other genes encoding such as: *Eos, GITR, CTLA4*, and simultaneously suppresses activation of: IL-2, IL-4 and INF-γ (1–4). Other important function-associated genes in Tregs are: *IL2RA* (CD25), *CTLA4* (CD152), *TNFRSF18* (GITR), *IKZF2* (Helios), *IKZF4* (Eos) and *Tet2* (5–8).

Tregs ability to prevent excessive immune response has been tested in many clinical trials. In human autoimmune diseases or transplantation, a broken tolerance can be restrained by administration of Tregs. In our hands, the therapy with expanded Tregs was successfully administered in type 1 diabetes or graft versus host disease (GvHD) after bone marrow transplantation (9–11). Until now, mainly polyclonal Tregs have been used in clinical therapies due to the problem in technical expansion of antigen-specific cells (12, 13). Only recently, we have developed a technique, which allows for efficient production of bulk quantities of antigen-specific Tregs, which seems to be a promising tool for autoimmune therapies (14). Our method is based on antigen-loaded monocytes which preferentially activate Tregs specific to presented antigen. Because our work is mainly focused on type 1 diabetes, we used either whole insulin or insulin β chain peptide 9-23 as antigens. Surprisingly, we have found that Tregs generated with β chain peptide 9-23 were significantly more suppressive than those generated with the whole insulin.

**Figure 1:**
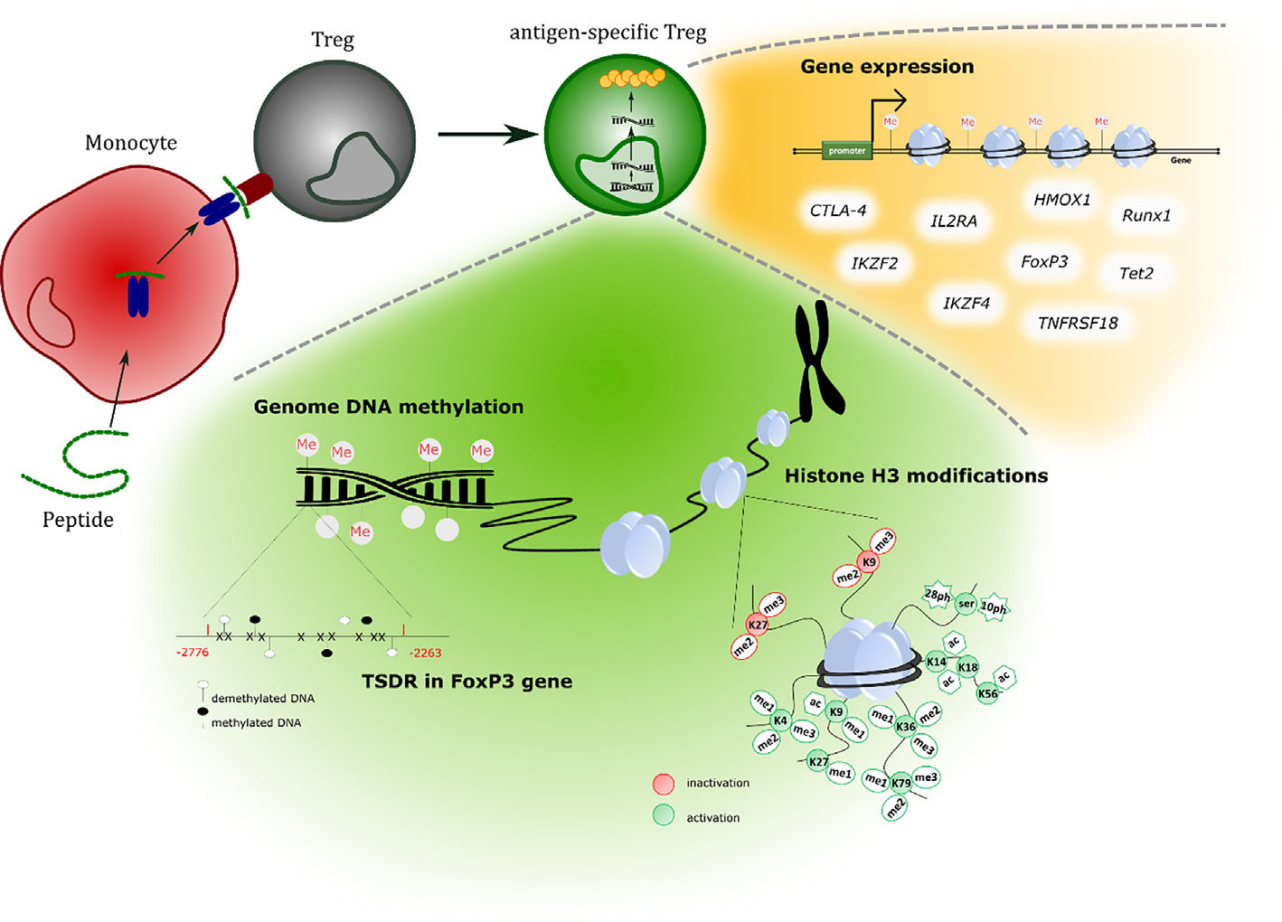
An overview of tests performed to study epigenetic features of generated cells.

Looking for the reasons of such a difference, we examined epigenetic features, presented at **Figure 1**, of both: Tregs (CD4^+^CD25^high/+^CD127^-^) and T effector cells (CD4^+^CD25^low/-^CD127^+^; Teffs) generated with monocytes loaded with either whole insulin or insulin β chain peptide 9-23 sorted as antigen-specific (index SPEC) cells. We have also looked at Tregs and Teffs unspecific to the antigens (index UNSPEC) as well as those expanded with anti-CD3/anti-CD28 beads used currently as the polyclonal (index POLY, 1:1 ratio bead:cell) in the treatment of type 1 diabetes. Taking into account already known TSDR-mediated regulation of *FoxP3* gene, we assumed that other epigenetic changes could be also very important in the activity of the manufactured cells and therefore we investigated global genomic DNA methylation, methylation in specific TSDR region of *FoxP3* gene and histone H3 post-translational modifications (PTMs). In addition, we assessed in all subsets the expression of genes crucial in the activity of Tregs, such as: *FoxP3, CTLA-4, IKZF2, IKZF4, IL2RA, TNFRSF18, Tet2, Runx1* and *HMOX1*. Indeed, we found significant differences between the subsets, which could impact the activity of the cells.

## Materials and Methods

### Research Material

Buffy coats, with unknown HLA, were obtained from the Regional Centre for Blood Donation and Treatment in Gdańsk from volunteers donating blood. All tests were conducted on male volunteers aged 18-65.

### Cells Preparation

**Figure 2:**
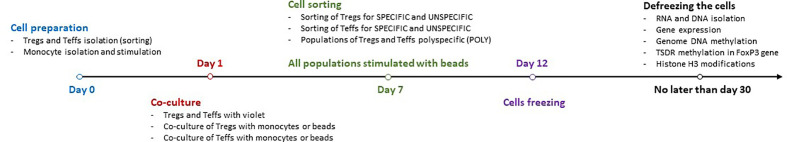
The scheme of obtaining cells and their further use.

Detailed procedure for cells preparation was described by Iwaszkiewicz-Grzes D. et al. previously (14). General procedure is presented at the workflow in **Figures 2** and **S1**.

Tregs and Teffs were freshly isolated from buffy coats obtained from anonymous healthy volunteer blood donors according to previously described protocol (15). Briefly, peripheral blood mononuclear cells (PBMCs) were isolated from buffy coats by gradient centrifugation using Ficoll-Paque Plus. Collected PBMCs were counted and separated into two tubes, first tube for isolation of CD4^+^ cells using negative immunomagnetic selection method (StemCell EasySep™ Human CD4 Negative Selection Kit, StemCell Technologies, Canada) and second tube for isolation of CD14+ cells using positive immunomagnetic selection method (StemCell EasySep™ Human CD14 Positive Selection Kit, StemCell Technologies, Canada).

CD4^+^ T cells were transferred into cell culture flask in X-VIVO culture medium with addition of penicillin/streptomycin and remained to the next day under standard conditions (37°C, 5% CO_2_, 95% O_2_). At the same time, CD14^+^ monocytes (Mo) were isolated according to the manufacturer’s instructions. Next, CD14^+^ cells were suspended in X-VIVO culture medium (Lonza), spread out into plates and stimulated for 24h with tested antigens: whole insulin (index INS, 350µg/well/ml (16), Actrapid^®^ Penfill^®^, Novo Nordisk A/S) and insulin β chain peptide (index B:9-23, 25μg/well/ml; Lipopharm; Gdansk, Poland) under standard conditions (37°C, 5% CO_2_, 95% O_2_).

After 24h CD4^+^ T cells were stained with monoclonal antibodies (mAbs): CD3, CD4, CD25 and CD127, and sorted with FACS AriaII sorter (BD Biosciences, USA) into Tregs (CD3^+^CD4^+^CD25^high/+^CD127^−/low^lin^−^doublet^-^) and Teffs (CD3^+^CD4^+^CD25^low/-^CD127^+^lin^−^doublet^-^).

Next, Tregs and Teffs were stained with violet (Cell Trace Violet Cell Proliferation Kit, Life Technologies, 1μM; 15min, 37°C) (17). At the same time, previously prepared monocytes were collected and irradiated.

We prepared the following conditions: Tregs/Mo stimulated with INS or B:9-23, Tregs/beads (ExpAct Treg Beads conjugated to CD28, Anti-Biotin, and CD3-Biotin monoclonal antibodies; MACS^®^GMP; in 1:1 ratio (bead:cell)), Teffs/Mo stimulated with INS or B:9-23 and Teffs/beads in X-VIVO culture medium with addition of IL2 (100 IU/ml), heat-inactivated human serum (10%) and penicillin/streptomycin. Cultures containing monocytes were additionally stimulated with anti-CD28 and anti-CD154 antibodies at a final concentration of 5μg/ml/well each (BD Pharmingen™ Purified NA/LE Mouse Anti-Human CD154/CD28). After 6 days of expansion cells were collected and sorted based on violet fluorescence (**Figure S1**) (17). Cells responding to the antigen presented by the monocytes were identified as antigen-specific (index SPEC), non-proliferating cells were identified as unspecific (index UNSPEC). During whole procedure cells were stimulated only once with monocytes. Cells stimulated with beads were treated as polyclonal (specific against many antigens, index POLY).

Obtained cells were expanded with beads (no longer than 5 days) in order to obtain enough cells for all tests, at least 1 million cells per condition (Tregs: POLY, SPEC, UNSPEC; Teffs: POLY, SPEC, UNSPEC) for cells stimulated with whole insulin and insulin β chain peptide 9-23 and stored in -70°C maximum 1 month.

### RNA Extraction and RT-qPCR

Total RNA was isolated using AllPrep^®^ DNA/RNA Mini Kit (Qiagen, USA) following the manufacturer’s instruction. Assessment of RNA concentration and purity was measured *via* spectrophotometer (Epoch, BioTek). Obtained RNA was stored in -70°C until use.

500ng total RNA was reverse transcribed into cDNA using High Capacity cDNA Reverse Transcription Kit (ThermoFisher Scientific) under standard conditions: step 1 - 25°C/10 min; step 2 - 37°C/120 min; step 3 - 85°C/5 min; step 4 - 4°C/∞ min. The expression of target genes, characteristic for Tregs: *FoxP3, IKZF2, IKZF4, IL2RA, TNFRSF18, Tet2, Runx1* and *HMOX1* was detected using FastStart Essential DNA Probes Master (Roche, Switzerland) on LightCycler^®^96 (Roche, Switzerland) in accordance to the manufacturer’s protocols in prepared cell populations (Tregs: POLY, SPEC, UNSPEC; Teffs: POLY, SPEC, UNSPEC). The primer sequences (Sigma-Aldrich) and used probes (Universal ProbeLibrary Set, Human with Probes; Roche, Switzerland) were designed using Assay Design Center Software (Roche) and are listed in **Table 1**. GAPDH was used as the housekeeping gene and the normalized expression ratio of the target genes in prepared cell populations was calculated using the 2^-ΔΔCt^ (Livak method) (18). All reactions were carried out in triplicate from three independent experiments.

**Table 1:** Sequences of primers used for real-time PCR.

Gene Name	RefSeq Accession Number	Primer	Amplicon Size (nt)	%GC	Tm	Primer Sequence (5’-3’) Forward	Probe #
GAPDH*	NM_001289745.1	Fw	70	45	60	CCCCGGTTTCTATAAATTGAGC	#75
		Rv		58	59	GGCTGACTGTCGAACAGGA	
FoxP3	NM_014009.3	Fw	102	55	59	ACACTGCCCCTAGTCATGGT	#25
		Rv		50	60	GAGCTGGTGCATGAAATGTG	
CTLA-4	NM_005214.4	Fw Rv	65	56 50	60 60	TGGGTCCCAGGGAAGTTT TGACCTTGTGTTCTACCTGGTG	#25
IKZF2	NM_016260.2	Fw	64	45	59	CATCACATTGCTTTGCCCTA	#61
		Rv		48	59	TCATCACTGTCAGAGAGAGGCTA	
IKZF4	NM_022465.3	Fw	68	45	60	TCAGGCATTTGTTGTGCAGT	#3
		Rv		53	59	AGGGAAAGGCAGATGCTGT	
IL2RA	NM_000417.2	Fw	73	55	59	CCAACTTCCCAGTTCAGGAG	#45
		Rv		44	59	GGGTAGAGTGTGTGTGTTGTGTATT	
TNFRSF18	NM_004195.2	Fw	92	61	59	ACCTGGGTCGGGATTCTC	#10
		Rv		61	59	CACAGCCAGTTGGACACG	
Tet2	NM_001127208.2	Fw	93	36	59	AAAGATGAAGGTCCTTTTTATACCC	#68
		Rv		48	59	ACCCTTCTGTCCAAACCTTTC	
Runx1	NM_001754.4	Fw	61	41	60	CCAAAGAGTGTGGAATTTTGGT	#55
		Rv		50	59	AAACAGGGCGAGTTGCAT	
HMOX1	NM_002133.2	Fw	61	55	59	CCCTTCAGCATCCTCAGTTC	#84
		Rv		58	59	GACAGCTGCCACATTAGGG	

*GAPDH was used as an endogenous control.

### Genomic DNA Extraction and Global DNA Methylation

DNA was isolated using AllPrep^®^ DNA/RNA Mini Kit (Qiagen, Germany) following the manufacturer’s instruction from following cells: Tregs: POLY, SPEC, UNSPEC; Teffs: POLY, SPEC, UNSPEC for cells stimulated with whole insulin and insulin β chain peptide 9-23. Assessment of DNA concentration and purity was measured *via* spectrophotometer (Epoch, BioTek). Obtained DNA was stored in -20°C until use.

Quantification of genomic DNA methylation was performed using Methylated DNA Quantification Kit (Colorimetric) (Abcam, UK). 100 ng/µl of DNA was used per reaction (well) under manufacturer’s instruction. Absorbance was read at 450 nm *via* Epoch (BioTek) spectrophotometer with Gene5 software. Obtained absorbance was used to calculate percentage of genomic DNA methylation in each cell population. The total/global amount of methylated DNA was calculated by generation of a standard curve. Next, the slope (OD/ng) of the standard curve was determined using linear regression and then the analysis of absolute/total quantification of 5-mC in total DNA was determined.

### Histone Extraction and Histone H3 Modification

Cells stimulated with beads, whole insulin or insulin β chain peptide 9-23 (Tregs: POLY, SPEC, UNSPEC; Teffs: POLY, SPEC, UNSPEC) were pelleted and histones isolated with Histone Extraction Kit (Abcam, UK) according to the manufacturer’s instructions. Two Assay Control Proteins were prepared with the final concentrations of 5ng/µl and 25ng/µl. 150ng of histone extract per well for each modification were used in triplicate. Assessment of histone concentration and quality were measured *via* spectrophotometer Epoch (BioTek). 21 histone H3 modifications, which include the most important and the most well characterized patterns, were measured using Histone H3 Modification Multiplex Assay Kit (Colorimetric, Abcam) and are listed in **Table 2**. Obtained absorbance was used to calculate % of individual histone H3 modifications.

**Table 2:** Histone H3 modifications.

Methylation	Acetylation	Phosphorylation
H3K4me1↑	H3K9ac↑	H3ser10ph↑
H3K4me2↑	H3K14ac↑	H3ser28ph↑
H2K4me3↑	H3K18ac↑	
H3K9me1↑	H3K56ac↑	
H3K9me2↓		
H3K9me3↓		
H3K27me1↑		
H3K27me2↓		
H3K27me3↓		
H3K36me1↑		
H3K36me2↑		
H3K36me3↑		
H3K79me1↑		
H3K79me2↑		
H3K79me3↑		

↑ activating modification.

↓ inactivating modification.

### DNA Bisulfite Conversion and Methylation-Specific PCR

Quantitative methylation-specific polymerase chain reaction with methylated (M) and unmethylated (U) primers was used for detection of methylation of the TSDR region in *FoxP3* gene. Briefly, genomic DNA was extracted from maximum 1 million of cells using AllPrep^®^ DNA/RNA Mini Kit (Qiagen, Germany) and submitted to bisulfite conversion using the EpiTect^®^ Bisulfite Kit (Qiagen, Germany) under manufacturer’s instruction. First, DNA bisulfite conversion was performed in which unmethylated cytosine residues are deaminated to uracil and methylated cytosine (5-mC) residues remain intact. 500 ng of isolated DNA per reaction were used. Bisulfite reaction was performed in the thermocycler with the following parameters: 95°C for 5 min, 60°C for 25 min, 95°C for 5 min, 60°C for 85 min, 95°C for 5 min and 60°C for 175 min. After conversion, DNA was subjected to quantitative methylation-specific PCR procedure using TB Green Premix Ex Taq II (Takara, Japan) on LightCycler^®^96 (Roche, Switzerland) in accordance with the manufacturer’s protocols. EpiTect^®^ PCR Control DNA Set (Qiagen, Germany) was used as positive and negative controls. Unconverted DNA was considered as a negative control. Methylated and unmethylated primers for *FoxP3* gene intron 1 were designed using MethPrimer 2.0 Software by Zafari et al. and are listed in **Table 3** (19, 20). Real-time PCR was performed in a final reaction volume of 20 μl using the Roche Life Science LightCycler^®^ 96 including 5 pmol of each forward and reverse methylated/demethylated primer and 50–100 ng of bisulfite-treated genomic DNA. PCR consisted of an initial denaturation at 95°C for 30 s, 40 cycles of denaturation at 95°C for 5 s, followed by 60°C for 30 s, 1 cycle of melting at 95°C for 5 s, followed by 60°C for 1 min, 1 cycle of cooling at 50°C for 30 s. The level of methylation was also verified by electrophoresis on a 2% agarose gel using 100 bp DNA Ladder (Invitrogen, USA) as a marker.

**Table 3:** Primers for TSDR analysis (19).

Primer set	Primer	Primer Sequence (5’-3’) Forward	PCR product size	Tm
demethylated	Fw Rv	GGATAGGGTAGTTAGTTTTTGGAATG CCACCATTAACATCATAACAACCA	117	62.6 64.1
methylated	Fw Rv	GATAGGGTAGTTAGTTTTCGGAAC CCGCCATTAACGTCATAACG	116	59.9 64.9

### Statistics

The Statistica 13.0 software was used to perform all statistical analysis. Significance was calculated using the *t-* test. Significant results are marked with * (p<0.05), ** (p<0.01) or *** (p<0.001).

## Results

### Influence of Antigen Stimulation on Gene Expression

**Figure 3:**
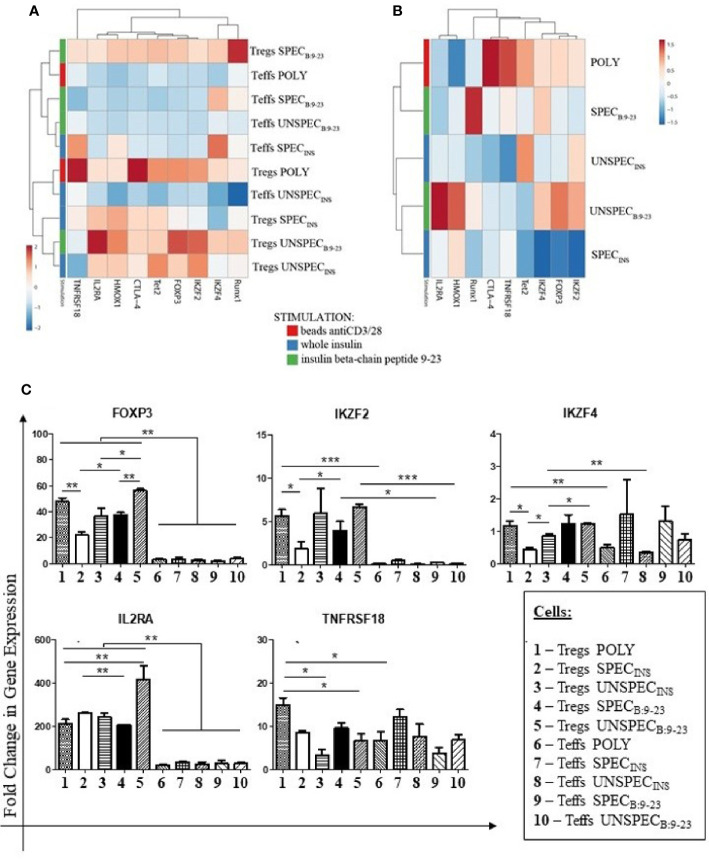
Fold Change in Gene Expression in Tregs and Teffs. **(A):** Clustering and heat maps analysis of gene expression in Tregs: POLY, SPEC, UNSPEC and Teffs: POLY, SPEC, UNSPEC. **(B)** Clustering and heat maps analysis of gene expression only in Tregs. **(C)** Graphs presenting changes in cells populations. Only the expressions in which statistical significance occurred are presented. The cells were stimulated with beads anti-CD3/anti-CD28 (index POLY) or monocytes loaded with whole insulin (index INS) or insulin β chain peptide 9-23 (index B:9-23). The cells responding to antigen have an index SPEC, cells not recognizing the antigen have an index UNSPEC. All stimulations were performed in triplicates (three tests for whole insulin and three tests for insulin β chain peptide 9-23). The results are presented as mean+/- SD. Significance was calculated using the t- test, significant results are marked with * (p<0.05), ** (p<0.01) or *** (p<0.001). Heat maps were prepared using ClustVis tool based on correlation distance and average linkage between clusters (21).

In the current study we selected several genes known to be involved in the function of Tregs and investigated their expression in particular subsets of cells. We compared polyclonal Tregs, which are a cellular medicinal product used in the treatment of type 1 diabetes, with Tregs specific and unspecific against whole insulin (index INS) or insulin β chain peptide 9-23 (index B:9-23). Polyclonal Tregs were treated as a reference for other cells. To obtain fully valuable results, we also performed the same tests for effector T cells: polyclonal, specific and unspecific against presented antigen. The obtained results are presented in **Figure 3**, and the p values of statistical significance in **Table S1**.

**Figure 3A** shows gene expression in all populations in a form of a heatmap. With the exception of the IKZF4 gene, Tregs showed higher expression of the studied genes than Teffs. Teffs SPEC_INS_ were the only ones among the effectors which showed low but noticeable expression of IKZF4, TNFRSF18 and HMOX1 genes. Among Tregs subsets (**Figure 3B**), Tregs SPEC_B:9-23_ and Tregs POLY were of special interest as they expressed all analyzed genes. Tregs SPEC_B:9-23_ showed the highest levels of expression of RUNX1 and IKZF4 genes but the expression of other genes was moderate. On the other hand, Tregs SPEC_INS_ were the cells with the lowest expression of IKZF2, FoxP3, IKZF4, Tet2 and RUNX1 genes. Tregs UNSPEC_INS_ also showed low expression of the majority of genes, especially IL2RA, HMOX1 and FoxP3, when compared to Tregs SPEC_B:9-23_.

We could observe statistically significant differences between particular subsets in the expression of five genes: FoxP3, IKZF2, IKZF4, IL2RA and TNFRSF18 which are presented separately in **Figure 3C**. Values of statistical significance for gene expression are presented in **Table S1**.

### The Influence of Antigen Stimulation on Genomic DNA Methylation

**Figure 4:**
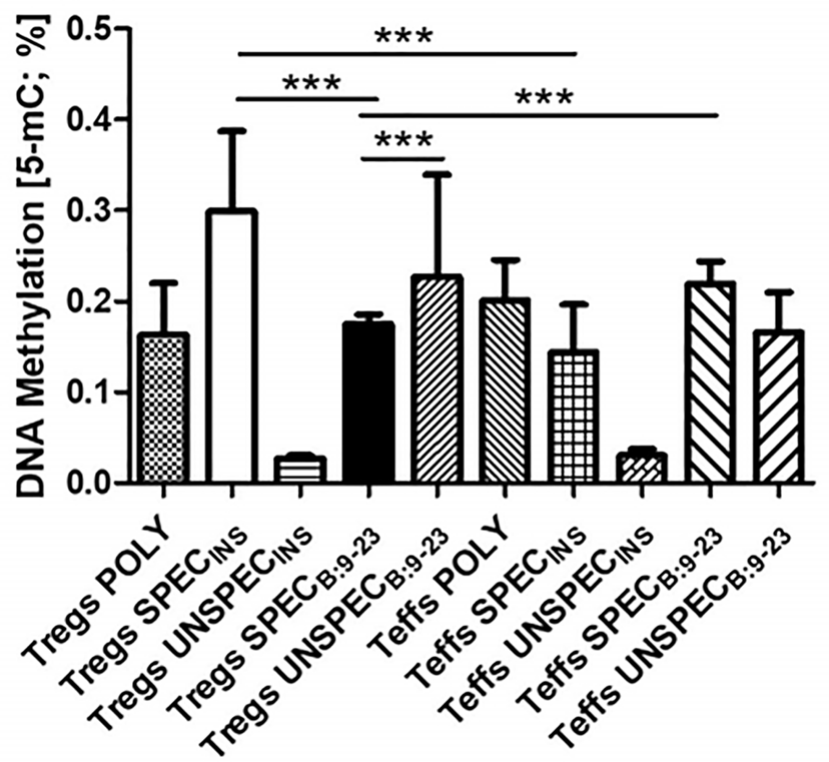
Global DNA methylation in Tregs and Teffs. Comparison of 5-methylocytosine (5-mC) production in Tregs: POLY, SPEC, UNSPEC and Teffs: POLY, SPEC, UNSPEC. During the tests cells were stimulated with beads anti-CD3/ant-CD28 (index POLY) or monocytes loaded with whole insulin (index INS) or insulin β chain peptide 9-23 (index B:9-23). Cells responding to antigen have an index SPEC, cells not recognizing the antigen have an index UNSPEC. All analyzes were performed for six tests (three tests for whole insulin and three tests for insulin β chain peptide 9-23) in triplicate. The results are presented as mean+/- SD. Significance was calculated using the t- test, significant results are marked with * (p<0.05), ** (p<0.01) or ***(p<0.001).

Based on a colorimetric assay for quantification of global DNA methylation by measuring levels of 5-methylocytosine (5-mC) we observed statistically significant differences between cells (**Figure 4**). Tregs SPEC_INS_ and Tregs UNSPEC_B:9-23_ had the highest level of methylation. Tregs SPEC_B:9-23_ were much less affected. Their methylation differed significantly from Tregs UNSPEC_B:9-23_ (t-test; p *<*0.0001), Tregs SPEC_INS_ (t-test; p *<*0.0001) and Teffs SPEC_B:9-23_ (t-test; p *<*0.0001). Tregs SPEC_B:9-23_ were also significantly different from antigen-specific Teffs stimulated with the same antigen (Teffs SPEC_B:9-23_; t-test; p<0.0001). We did not observe significant differences between Tregs POLY and other cells.

### The Influence of Antigen Stimulation on TSDR Methylation in FoxP3 Gene

**Figure 5:**
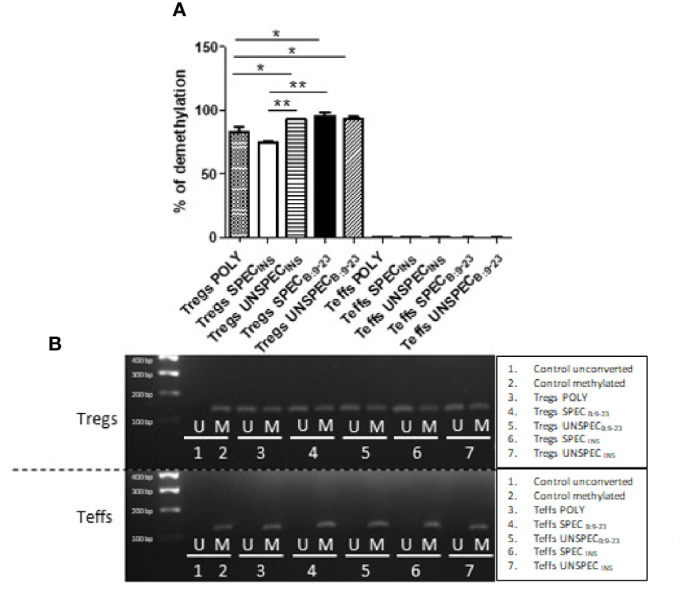
FoxP3 demethylation in TSDR region in Tregs and Teffs. **(A)** Comparison of percentage of demethylation in Treg-specific demethylated region (TSDR) in Tregs: POLY, SPEC, UNSPEC and Teffs: POLY, SPEC, UNSPEC. **(B)** Representative results showing FoxP3 demethylation in all cell cultures was detected by agarose gel electrophoresis [reaction with methylated (M) and unmethylated (U) primer]. During the tests cells were stimulated with anti-CD3/anti-CD28 beads (index POLY) or monocytes loaded with whole insulin (index INS) or insulin β chain peptide 9-23 (index B:9-23). Cells responding to the antigen have an index SPEC, cells not recognizing presented antigen have an index UNSPEC. All analyzes were performed for six tests (three tests for whole insulin and three tests for insulin β chain peptide 9-23) in triplicate. The results are presented as mean+/- SD. Significance was calculated using the t- test, significant results are marked with *(p<0.05), **(p<0.01) or ***(p<0.001).

We next used quantitative methylation-specific polymerase chain reaction with methylated and unmethylated primers for detection of methylation in TSDR region of FoxP3 gene. TSDR in all Tregs showed a level of demethylation over 75%, which was significantly more demethylated than TSDR of Teffs (t-test; p<0.0001) (**Figure 5A**). These results were also confirmed by agarose gel electrophoresis (**Figure 5B**). Tregs SPEC_INS_ showed the lowest level of demethylation (75%) among Tregs subsets. It was significantly less compared to Tregs UNSPEC_INS_ (t-test; p=0.0020) and Tregs SPEC_B:9-23_ (t-test; p=0.0065). Tregs POLY, whose demethylation was ≈80%, were significantly less demethylated than each of the three subsets of Tregs: Tregs SPEC_B:9-23_ (t-test; p=0.0229), Tregs UNSPEC_B:9-23_ (t-test; p=0.0433) and Tregs UNSPEC_INS_ (t-test; p=0.0451). All these three remaining Treg subsets showed demethylation over 90%.

### The Influence of Antigen Stimulation on Histone H3 Modifications

**Figure 6:**
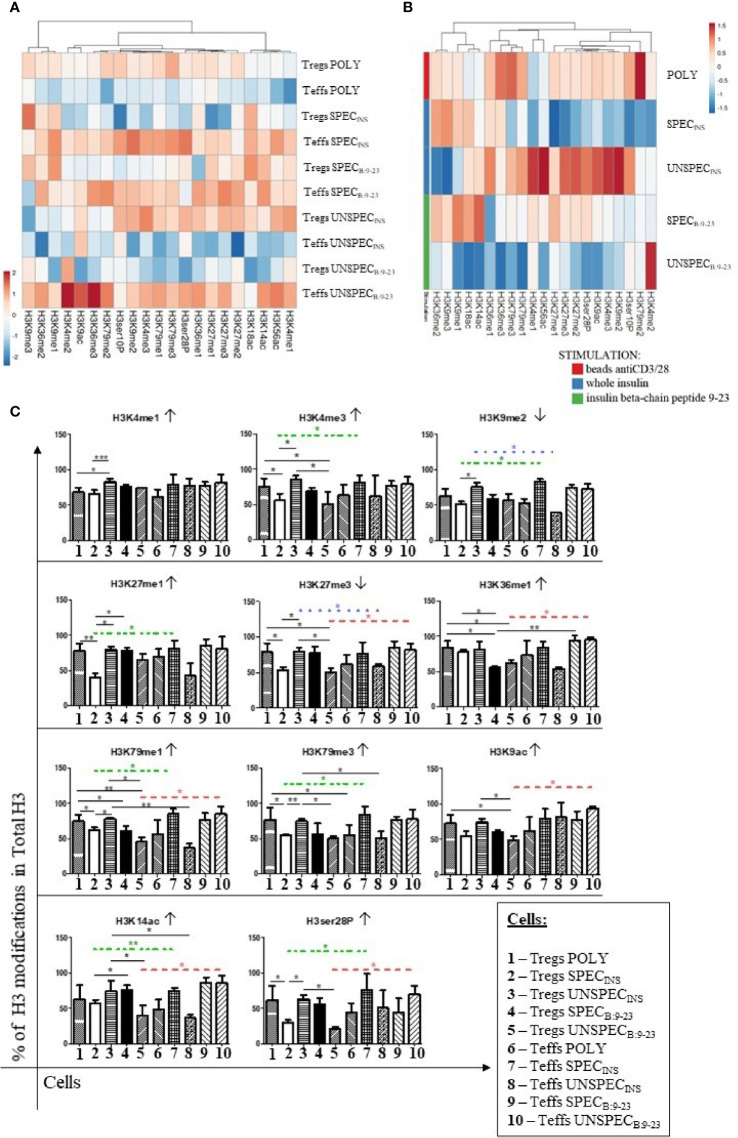
Percentage of histone H3 modification in total H3 in Tregs and Teffs. **(A)** Comparison of percentage of modifications in Tregs: POLY, SPEC, UNSPEC and Teffs: POLY, SPEC, UNSPEC. **(B)** Comparison of modifications only in Tregs. Cells were stimulated with anti-CD3/anti-CD28 beads (index POLY) or monocytes loaded with whole insulin (INS) or insulin β chain peptide 9-23 (B:9-23). **(C)** Graphs presenting changes in cells populations. Only modifications in which statistical significance occurs are presented. Cells responding to the antigen have an index SPEC, cells not recognizing the antigen have an index UNSPEC. All analyzes were performed for six tests (three tests for whole insulin and three tests for insulin β chain peptide 9-23) in triplicate. The results are presented as mean+/- SD. Significance was calculated using the t- test, significant results are marked with * (p<0.05), ** (p<0.01) or *** (p<0.001). Heat maps were prepared using ClustVis tool based on correlation distance and average linkage between clusters (21). ↑ - activating modification; ↓ - inactivating modification; **··­··** difference between specific Tregs and Teffs stimulated with insulin; **····** difference between unspecific Tregs and Teffs stimulated with insulin; **··­·· ­­­** difference between unspecific Tregs and Teffs stimulated with peptide 9-23.

The total concentration of histone H3 protein and individual modifications in ng was calculated and compared at heat-maps (**Figure S2**). Then, based on Total H3, the percentage of different cell modifications was determined (**Figure 6**). We analyzed gene activating modifications (↑) such as methylation: H3K4me(1-3), H3K9me1, H3K27me1, H3K36me(1-3), H3K79me(1-3); acetylation: H3K9ac, H3K14ac, H3K18ac, H3K56ac; phosphorylation: H3ser28P, H3ser10P and gene inactivating modifications (↓) such as methylation: H3K9me2, H3K9me3, H3K27me2, H3K27me3 (**Table 2**). All cultures were performed in triplicates.

The comparison of the precentage of histone H3 modifications in total H3 between Tregs and Teffs (**Figure 6A**) confirmed that the populations of Tregs showed opposite pattern of modifications than Teffs. Teffs, mainly Teffs SPEC_B:9-23_, Teffs SPEC_INS_ and Teffs UNSPEC_B:9-23_, were characterized by a relatively high level of modifications.

While studying the differences between differently stimulated Tregs (**Figure 6B**), Tregs POLY exhibited the highest level of histone modification, when compared to monocyte stimulated cells. When comparing antigen-specific subsets, we could observe that Tregs SPEC_INS_ showed a lower level of modification than Tregs SPEC_B:9-23_, excluding H3K18ac, H3K9me1, H3K9me3 and H3K36me2.

Interestingly, when compared to Teffs, Tregs were characterized by a relatively lower level of PTMs but it varied in respective Tregs/Teffs pairs (**Figure 6C**, **Table 4**). Importantly, whenever the difference in PTMs within Tregs/Teffs pair was significant, it was always low in one subset from the pair and always high in the other. The level of modifications in Tregs SPEC_INS_ was significantly lower than that in Teffs SPEC_INS_ in 7 out of 11 regions, in which any significant differences occurred (H3K4me3, H3K9me2, H3K27me1, H3K79me1, H3K79me3, H3K14ac and, H3ser28P). At the same time, there was no single modification, which level was different between Tregs SPEC_B:9-23_ and Teffs SPEC_B:9-23_ (**Figure 6C**). Tregs UNSPEC_INS_ showed a significantly higher degree of modifications than Teffs UNSPEC_INS_ in 5 out of 11 regions, in which any significant differences occurred (H3K9me2, H3K27me3, H3K79me1, H3K79me3 and, H3K14ac) (**Figure 6C**). The level of modifications in Tregs UNSPEC_B:9-23_ was significantly lower than that in Teffs UNSPEC_B:9-23_ in 6 out of 11 regions, in which any significant differences occurred (H3K27me3, H3K36me1, H3K79me1, H3K9ac, H3K14ac and, H3ser28P). Interstingly, H3K79me1 was the only one modified region, in which the significant differences were found between all three respective Tregs/Teffs pairs.

**Table 4:** Selectivity index (SI) for histone modifications.

Histone modification	SI [Teffs/Tregs]				
	POLY	SPEC_INS_	UNSPEC_INS_	SPEC_B:9-23_	UNSPEC_B:9-23_
**H3K4me1**	0,90	1,22	0,93	1,02	1,11
**H3K14ac**	0,78	**1,31**	0,49	1,13	**2,16**
**H3K56ac**	0,81	1,22	0,72	1,05	1,56
**H3K4me3**	0,84	**1,44**	0,73	1,12	1,59
**H3K9me2**	0,83	**1,64**	**0,51**	1,28	1,28
**H3K27me1**	0,89	**2,00**	0,53	1,08	1,24
**H3K27me3**	0,79	1,43	**0,75**	1,09	**1,62**
**H3K36me2**	0,78	1,07	0,60	1,06	1,37
**H3K79me1**	0,75	**1,37**	0,47	1,26	**1,86**
**H3K79me3**	0,70	**1,56**	0,67	1,35	1,56
**H3ser28P**	0,72	**2,55**	0,80	0,79	**3,30**
**H3K4me2**	0,92	1,16	0,82	0,98	1,10
**H3K9me1**	0,79	1,11	0,82	0,93	1,40
**H3K9me3**	0,95	0,97	1,07	0,83	1,19
**H3K27me2**	0,80	0,98	0,61	1,14	1,12
**H3K36me1**	0,88	1,08	0,67	1,69	**1,53**
**H3K36me3**	0,93	1,13	1,04	1,19	1,30
**H3K79me2**	0,75	1,18	0,87	1,11	1,27
**H3K9ac**	0,84	1,48	1,08	1,29	**1,93**
**H3K18ac**	0,91	1,02	0,91	0,90	1,36
**H3ser10P**	0,79	1,51	0,88	1,28	1,14

The selectivity index was calculated according to formula $SI=\frac{Teffs}{Tregs}$, where:

Teffs – percentage of histone H3 modification in total H3 in T effector cells.

Tregs – percentage of histone H3 modification in total H3 in T regulatory cells.

green - difference between specific Tregs and Teffs stimulated with insulin;

blue - difference between unspecific Tregs and Teffs stimulated with insulin;

red - difference between unspecific Tregs and Teffs stimulated with peptide 9-23.

Modifications with significant differences are shown in **Figure 6C** and p values in **Table S2**. Modifications without correlations are presented in supplementary materials at **Figure S3**.

## Discussion

In this study we aimed to examine the epigenetic background of the difference in the activity between Tregs expanded with monocytes loaded with different peptides, such as whole insulin or insulin β chain peptide 9-23. We have found different pattern of histone PTMs and different level of DNA methylation as well as different expression of genes crucial for Tregs development and suppressive function between the subsets expanded with these different antigens.

Until now, polyclonal T regulatory cells have been used in many clinical trials as a potent medicinal product that downregulates immune response during autoimmune diseases (9). Polyclonal cells, obtained by anti-CD3 and anti-CD28 stimulation, exert positive effect on patients with type 1 diabetes and significantly reduce the inflammatory response (16). One attempt to improve this therapy is to use specific Tregs directed toward disease-causing antigens. Such Tregs should traffic only into the inflamed tissue and suppress autoreactive lymphocytes *in situ* by response against specific antigens. Such an antigen-specific preparation may improve effectiveness of the currently administered treatment with polyclonal Tregs, reduce the required dose and limit adverse effects related to the interaction of Tregs with distant unrelated tissues. In our previous study, we proved that Tregs responding to a particular antigen showed higher potency to suppress Teffs than polyclonal cells. Antigen-specific Tregs retained a higher level of FoxP3^high^ expression and also maintained suppressive phenotype, which makes them more potent to surpass the excessive immune response during autoimmunity (14). Interestingly, Tregs specific to insulin β chain peptide 9-23 were more suppressive than those generated with whole insulin. We found that antigen-specific Teffs could be generated with monocytes loaded with antigens, too. These results are important as they confirmed our *in vivo* data from type 1 diabetes in which we found that the disease-specific antigens can induce both specific Tregs and Teffs and the balance between these two subsets might be associated with the course of the disease (22). Importantly, current report confirms *in vitro* that the whole insulin is a poor stimulator of Tregs and the efficient induction of tolerance should be performed with other peptides, like β chain peptide 9-23.

It is highly interesting which epigenetic changes are exerted by the particular stimuli. It is widely known that the sustained suppressive phenotype of Tregs requires progressive demethylation in Tregs-specific signature genes (23). The majority of the studies focus on *FoxP3* gene and its regulation, because *FoxP3* is a master regulator that provides Tregs function and ensures phenotype maintenance. However, recent data shows that *FoxP3* expression alone is unable to preserve Tregs function without acquisition of Treg-specific epigenome. It is well-established that Tregs deprived of CNS2 or Tregs with high TSDR methylation lack *FoxP3* expression and suppressive function and could even acquire abilities to produce pro-inflammatory cytokines (24, 25). TSDR demethylation within the first intron of *FoxP3* gene locus is specific for Tregs, while in Teffs this region is highly methylated. The analysis of methylation status is reliable and correlates with the generation of stable Tregs (26, 27). The state of demethylation is needed for binding with other transcription factors such as: CREB, NFAT, RUNX to enable *FoxP3* expression (28). The research conducted by Miyao et al. (29) has revealed that TSDR demethylation acts as epigenetic memory that provides linage stability, even in the environment that contributes to *FoxP3* downregulation and thus indicate that stable CNS2 ensures Tregs persistence. In a similar study on effectiveness of antigen-specific Tregs obtained after stimulation with APCs, scientists confirmed that antigen-specific Tregs possessed a comparable average demethylation level (range 70,1-95.2%) to polyclonal cells, while specific and polyclonal Teffs had less than 0,2% of demethylation (30). For these reasons, the measurement of TSDR demethylation was applied as a useful quality control tool in the manufacturing of expanded polyclonal Tregs product (15, 31). In our research, all Tregs showed demethylation over 75**%** and Teffs were almost 100% methylated, which indicates that during cell culture all Tregs remained stable. Worth emphasizing are Tregs SPEC_B:9-23_, whose demethylation was significantly higher than Tregs SPEC_INS_ and POLY, which confirms their usefulness as a drug candidate superb to polyclonal Tregs.

The process of genomic DNA methylation is rapid and flexible during T cell activation and differentiation (32). 5**’**-methylcytosine (5-mC) depletion is a hallmark of active transcription and it is involved in determination of lymphocyte function (33). In our study we examined total percentage of DNA methylation in T lymphocytes and we saw various pattern of methylation in particular subsets. Nevertheless, Tregs SPEC_B:9-23_ were significantly less methylated than Tregs SPEC_INS_. We performed correlation analysis between global DNA methylation and TSDR methylation, and we did not observe any statistical significance. However, we can notice a trend toward increased DNA methylation and diminished TSDR demethylation in Tregs SPEC_INS_, and low global methylation and substantial TSDR demethylation in Tregs UNSPEC_INS._ Moreover, higher % of total DNA methylation does not affect TSDR demethylation in Tregs UNSPEC_9-23_.

Histone PTMs play a major role in chromatin remodeling, due to changes in electrostatic charge of histone protein tails, and creation of docking sites for proteins containing bromodomains or chromodomains that recognize acetylated or methylated lysine, respectively (34). The term **“**histone code**”** is used to describe the influence of histone modifications on gene expression and indicates that histone machinery decides which part of gene is transcribed (35). Genome-wide studies have revealed that different regions have distinct histone-modifications patterns, enabling expression of specific class of genes. A large number of studies contributed to understanding the functions of individual histone modifications. And thus, acetylation of lysine residues, is believed to be enriched in highly active promoters and increase transcription. Lysine: 4, 36, 79 mono-, di-, tri-methylation and 9, 27 mono-methylation is associated with active genetic status. Conversely, lysine: 9, 27 di- and tri-methylation is a repressive mark, resulting in gene inactivation. In turn, H3ser10P is responsible for gene activation and, like H3ser28P, for chromosome condensation during mitosis (36, 37). Histone modifications can alter as a result of activation process in CD4+ cells. Lamere SA. et al. (38) have revealed, that upon CD4+ activation, the dynamics of H3K4 methylation in promoter varies, and matches the RNA expression. Many differences in histone H3 methylation have been observed in gene promotors between Tregs and Teffs (39). In our study we decided to measure 21 histone H3 modifications, which consisted of lysine: 4, 9, 27, 36, 79 methylation, serine: 10, 28 phosphorylation and lysine: 9, 14, 18, 56 acetylation. We conclude that the type of stimulation (whole insulin or insulin β chain peptide 9-23) has an impact on PTMs.

The process of histone alteration is dynamic upon environmental conditions and is believed to be an indicator of gene activation status (40). The study of Th1 and Th2 differences in histone modifications in crucial gene signatures confirms the presence of active marks in given cell population with repressive histone marks in opposing cell line (41, 42). Moreover, substantial differences were not found between Tregs and conventional T cells based on H3K4me4 and H3K27me3 modifications (39). In our study we also did not see many differences between polyclonal Tregs and Teffs, except H3K79me3, with a predominance in Tregs (**Figure 6A**). However, upon antigen stimulation we observed changes in histone H3 modifications. In general, permissive H3 modifications (H3K4me1, H3K4me3) are abundant in indispensable regions such as: *FoxP3* promoter and intronic enhancer elements, and are connected with active promoters of up-regulated genes (*IL2RA, CTLA4, TNFRSF18, FOLR4*) (28, 43). All the above allow to maintain stable *FoxP3* expression and cell lineage commitment. In our study, Tregs SPEC_INS_ were the least modified of H3K4me1/3, but high amount of such modifications was seen in all Tregs subsets, with the predominance of Tregs UNSPEC_INS_ (**Figure 6A**). Another important modification regarding Tregs is H3 acetylation by histone acetyltransferases (HATs) CBP and p300. It allows proper development and maintenance of the suppressive function of Tregs. Upon activation HATs mediate acetylation of Tregs-related genes permitting their stable function. Disruption of p300 causes Tregs instability and promotes autoimmunity (44). Our research has revealed, that Tregs SPEC_9-23_ were more enriched in lysine 9,14,18,56 acetylation compared to Tregs SPEC_INS_ (**Figure 6B**). Regarding H3K27 methylation, the EZH2 methyltransferase contributes to cell stability and normal function.

It might be connected with the closed chromatin state in genes that are down-regulated by *FoxP3* (45–47). Moreover, EZH2 disruption leads to Tregs impairment and strengthens the anti-tumor immunity (48), which indicates the prominent role of H3K27me3 in Tregs. Here, we saw that Tregs SPEC_9-23_ and Tregs UNSPEC_INS_ have higher % of H3K27me3 modification than Tregs UNSPEC_9-23_ and Tregs SPEC_INS_ (**Figure 6A**).

Interestingly, we found a characteristic pattern related to the kind of stimulation in particular Treg/Teff pairs. Namely, a low level of PTMs in one subset from the pair was always associated with high level of PTMs in the other. This trend was found in the cells specific to insulin where low level of PTMs in Tregs SPEC_INS_ was associated with high level of PTMs in Teffs SPEC_INS_ and high level of PTMs in Tregs UNSPEC_INS_ was associated with low level of PTMs in Teffs UNSPEC_INS_. According to global DNA methylation there is an interdependence between low methylation level and high abundance of histone modifications in Tregs UNSPEC_INS_ and Tregs SPEC_9-23_, and decreased H3 modifications in Treg SPEC_INS_ and Tregs UNSPEC_9-23_ in relation to higher percentage of DNA methylation. But this trend did not occur in Teffs subset. There was no difference in Tregs**/**Teffs SPEC_B:9-23_ pair and the low level of modifications in Tregs UNSPEC _B:9-23_ was associated with the high one in Teffs UNSPEC _B:9-23_. The PTMs in which the differences were significant overlapped only partially between particular Tregs/Teffs pairs, which suggests that the stimulation with different peptides differently influenced PTMs. Nevertheless, mainly activating PTMs, such as H3K18ac, H3K9me1 and, H3K36me2 were modified in Tregs SPEC_B:9-23_ and Tregs SPEC_INS_ and additionally H3K14ac and H3K27me1 were modified only in Tregs SPEC_B:9-23_. It is also important to note that selectivity index (SI) is almost always below 1 for POLY and UNSPEC_INS_ Teffs/Tregs pairs (Tregs more modified than Teffs) and above 1 in other pairs (Tregs less modified than Teffs) (**Table 4**).

Gene expression analysis confirmed that antigen stimulation did not deprive Tregs of the expression of crucial genes. All Tregs subsets had high expression of genes (*FoxP3, IKZF4, IKZF2, CTLA4, IL2RA*) needed for their function and phenotype maintenance. Tregs SPEC_B:9-23_ were characterized by the highest expression but there was not much difference between Tregs POLY and Tregs SPEC_B:9-23._ On the other hand, the analysis has revealed diminished gene expression in Tregs SPEC_INS_. It is known, that *TNFRSF18* (GITR) and *IKZF4* (Eos) are constitutively expressed by FoxP3^+^ cells and their expression in FoxP3- cells increases during activation (49, 50). In our study, we noticed that Teffs SPEC_INS_ and SPEC_B:9-23_ had high or moderate expression of GITR and Eos, respectively, which confirms a state of activation upon antigen stimulation in these cells. Despite the high level of *FoxP3* mRNA expression, we did not see a correlation between high percentage of FoxP3^high^ cells, presented by Iwaszkiewicz-Grzes et al. (14), and mRNA level of *FoxP3*. Accordingly, other study also confirmed a modest relationship between protein levels and mRNA *FoxP3* expression, indicating a presence of other mechanisms involved in *FoxP3* expression (51). Bjur et al. (52) discovered that mRNA levels may not correlate with corresponding proteins due to post-transcriptional modifications. They noticed that, upon cell activation, changes in translational activity of specific mRNAs occur. Moreover, a recent study suggested that FoxP3 protein is subject to PTMs, which can alter its function, or even its stability (53).

Besides Foxp3 analysis, on the seventh day in our previous study (14), we performed phenotype test of: IKZF2, CD25 and CTLA-4, and observed high expression of each of them. The data on Helios and IL2RA are already published (14). Our current study has shown high percentage of relative gene expression of *CTLA4* in Tregs compared to Teffs. Nevertheless, Teffs cells had detectable *CTLA4* mRNA. At the protein level on day 7 after cell stimulation, Tregs and Teffs CTLA-4 expression on the cell surface was around 95% and 85% respectively. It is confirmed that CTLA-4 is continuously expressed on Tregs and occurs in T effector cells after activation, with maximum peak in proliferating, dividing cells (54). The discrepancies between low mRNA level at day 12 in Teffs and surface protein abundance can be explained by the CD4 activation model. Following TCR stimulation *CTLA4* mRNA is detected after 1 h with its peak around 24-36 h, and depends on mRNA half-life which is within the range from 4,6 h to 8,9 h, according to cell stimulation (55). Furthermore, Chan V. et al. conducted a study on CD4+ cells and observed an increase of mRNA *CTLA4* after 1 h after stimulation, maintained until 18 hours (56). Worth emphasizing is that CTLA-4 surface expression is modulated by many factors including TCR stimulation strength and depends on other mechanisms like CTLA-4 internalization and recycling (57).

Collectively, our results clearly demonstrate that stimulation with antigen-loaded monocytes presenting whole insulin or insulin β chain peptide 9-23 exerts epigenetic changes in Tregs. The type of stimulation determines the level of alterations in global DNA methylation pattern, and specific methylation of TSDR region as well as histone H3 PTMs. Insulin β chain peptide 9-23 promotes mainly Treg-oriented changes, while the phenotype after whole insulin stimulation was less clear. Hence, the pattern of the epigenetic changes may help finding the peptides that shape exclusively Tregs-mediated suppressive response or Teffs-mediated inflammatory response in future cellular drugs. Our observations indicate that antigen-specific Tregs during cell culture remained stable and comprise all Tregs-related features. It strengthens our confidence that our protocol allowing to obtain antigen-specific Tregs is a promising strategy of cell therapy, e.g. in type 1 diabetes.

## Data Availability Statement

The datasets presented in this study can be found in online repositories. The names of the repository/repositories and accession number(s) can be found below: Zenodo and 4442316 doi: 10.5281/zenodo.4442316.

## Ethics Statement

Ethical review and approval was not required for the study on human participants in accordance with the local legislation and institutional requirements. Written informed consent for participation was not required for this study in accordance with the national legislation and the institutional requirements.

## Author Contributions

Conceptualization, DI-G, MG and PT. Methodology, DI-G, MP, MG, ZU-W and PT. Software, DI-G. Validation, DI-G and MP. Formal analysis, DI-G, MP and MG. Investigation, DIG and PT. Writing—original draft preparation, DI-G, MP and MG. Writing—review and editing, PT. Visualization, DI-G. Supervision. PT. Project administration, DI-G and PT. Funding acquisition, DI-G and PT. All authors contributed to the article and approved the submitted version.

## Funding

This research was funded by National Centre for Research and Development (Poland), grant number LIDER/160/L-6/14/NCBR/2015 and STRATEGMED1/233368/1/NCBR/2014. ZUW was supported by “International Centre for Cancer Vaccine Science” project carried out within the International Research Agendas Programme of the Foundation for Polish Science co-financed by the European Union under the European Regional Development Fund.

## Conflict of Interest

The authors declare that the research was conducted in the absence of any commercial or financial relationships that could be construed as a potential conflict of interest.
