# Supplementary material for: Antigenic Challenge Influences Epigenetic Changes in Antigen-Specific T Regulatory Cells
Source: Front Immunol. 2021 Mar 23;12:642678. doi: 10.3389/fimmu.2021.642678 (PMC8044853; doi:10.3389/fimmu.2021.642678)
Supplement: Supplementary file 1 [file Presentation_1.pdf]

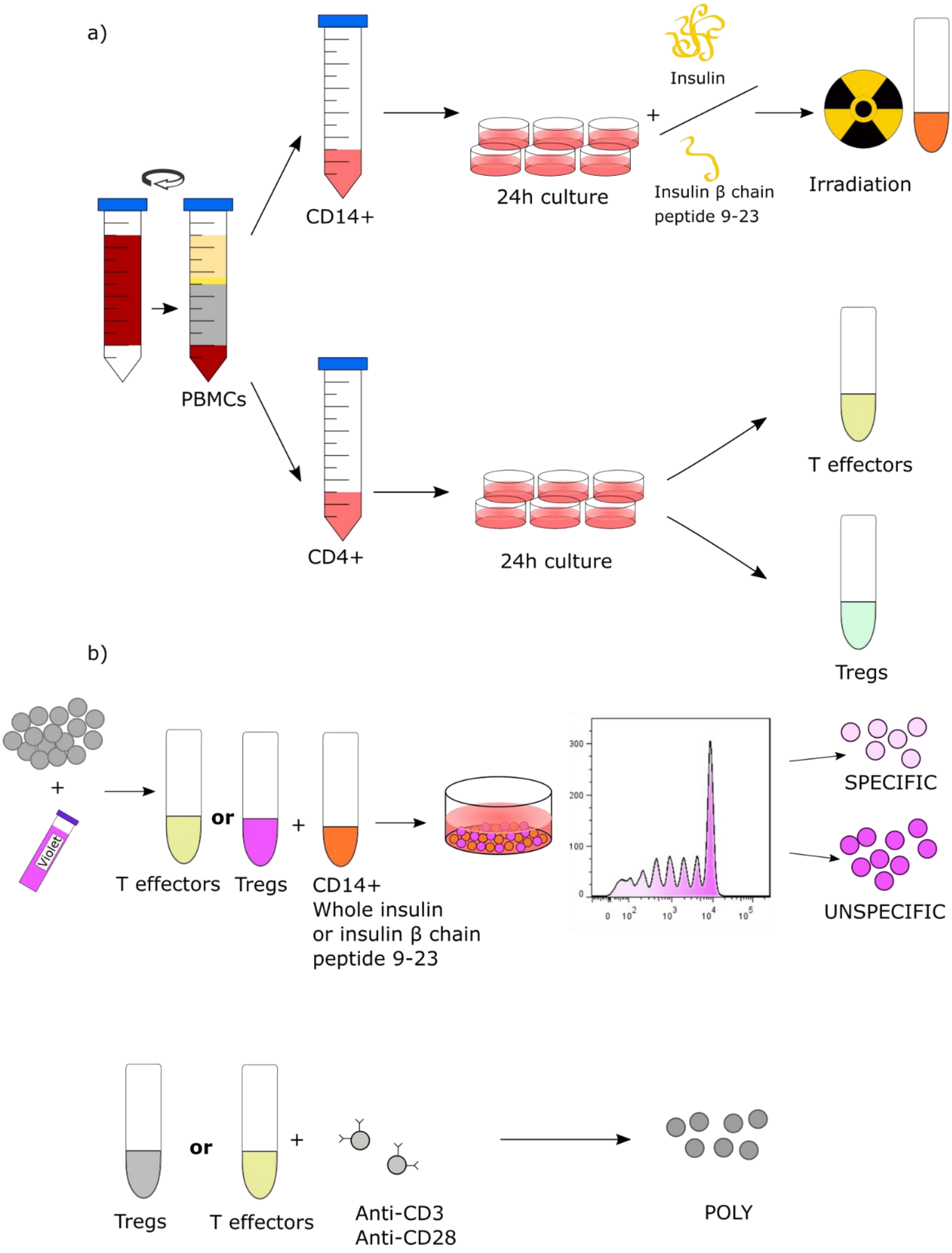

Figure S1. Workflow of cell preparation.

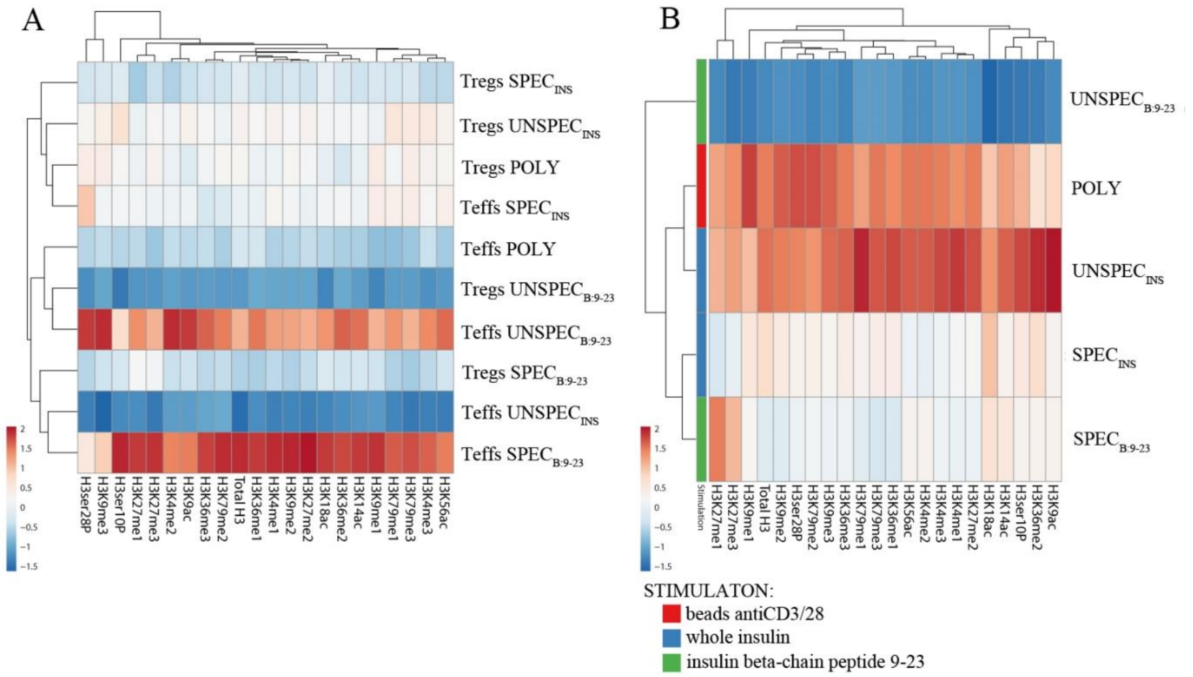

Figure S2. Histone H3 modifications and total H3 [ng/µg protein] in Tregs and Teffs.

A: Comparison of modifications in Tregs: POLY, SPEC, UNSPEC and Teffs: POLY, SPEC, UNSPEC. **B:** Comparison of modifications only in Tregs. Cells were stimulated with beads anti-CD3/ant-CD28 (index POLY) or monocytes loaded with whole insulin (INS) or insulin beta chain peptide 9-23 (B:9-23). Cells responding to antigen have an index SPEC, cells not recognising the antigen have an index UNSPEC. All analyzes were performed for six tests (three tests for whole insulin and three tests for insulin beta chain peptide 9-23) in triplicate. Heat maps were prepared using ClustVis tool based on correlation distance and average linkage between cluste [32]

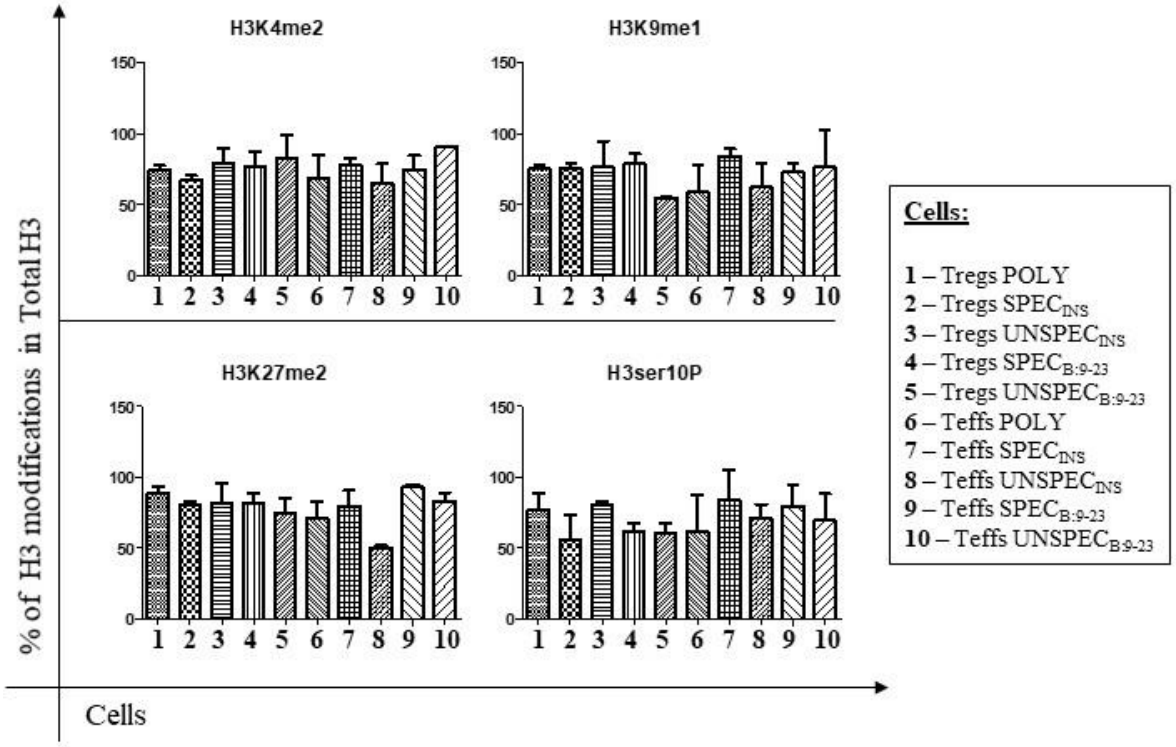

Figure S3. Percentage of histone H3 modification in total H3 in Tregs and Teffs.

Graphs presenting changes in cells populations without statistical significance between populations. Cells were stimulated with beads anti-CD3/ant-CD28 (index POLY) or monocytes loaded with whole insulin (INS) or insulin β chain peptide 9-23 (B:9-23). Cells responding to antigen have an index SPEC, cells not recognising the antigen have an index UNSPEC. All analyzes were performed for six tests (three tests for whole insulin and three tests for insulin β chain peptide 9-23) in triplicate. The results are presented as mean+/- SD. Significance was calculated using the t- test, significant results are marked with * (p<0.05), ** (p<0.01) or *** (p<0.001).
