# Supplementary material for: Antigenic Challenge Influences Epigenetic Changes in Antigen-Specific T Regulatory Cells
Source: Front Immunol. 2021 Mar 23;12:642678. doi: 10.3389/fimmu.2021.642678 (PMC8044853; doi:10.3389/fimmu.2021.642678)
Supplement: Supplementary file 2 [file Table_1.docx]

**Table S1.** Values of statistical significance for gene expression. Correlations not presented in the table did not show statistical significance.

| **Gene** | **Compared cell populations** | **P value** |
| --- | --- | --- |
| FOXP3 | Tregs POLY vs. Tregs SPEC_INS_  Tregs SPEC_INS_ vs. Tregs SPEC_B:9-23_  Tregs SPEC_B:9-23_ vs. Tregs UNSPEC_B:9-23_  Tregs UNSPEC_INS_ vs. Tregs UNSPEC_B:9-23_  Tregs POLY vs. Teffs POLY  Tregs SPEC_INS_ vs. Teffs SPEC_INS_  Tregs UNSPEC_INS_ vs. Teffs UNSPEC_INS_  Tregs SPEC_B:9-23_ vs. Teffs SPEC_B:9-23_  Tregs UNSPEC_B:9-23_ vs. Teffs UNSPEC_B:9-23_ | 0.0029  0.0253  0.0051  0.0332  <0.0001  0.0029  0.0061  0.0001  <0.0001 |
| IKZF2 | Tregs POLY vs. Tregs SPEC_INS_  Tregs SPEC_INS_ vs. Tregs SPEC_B:9-23_  Tregs POLY vs. Teffs POLY  Tregs SPEC_B:9-23_ vs. Teffs SPEC_B:9-23_  Tregs UNSPEC_B:9-23_ vs. Teffs UNSPEC_B:9-23_ | 0.0166  0.0195  <0.0001  0.0297  <0.0001 |
| IKZF4 | Tregs POLY vs. Tregs SPEC_INS_  Tregs SPEC_INS_ vs. Tregs UNSPEC_INS_  Tregs UNSPEC_INS_ vs. Tregs UNSPEC_B:9-23_  Tregs POLY vs. Teffs POLY  Tregs UNSPEC_INS_ vs. Teffs UNSPEC_INS_ | 0.0364  0.0255  0.0247  0.0032  0.0034 |
| IL2RA | Tregs POLY vs. Tregs SPEC_B:9-3_  Tregs SPEC_INS_ vs. Tregs SPEC_B:9-23_  Tregs POLY vs. Teffs POLY  Tregs SPEC_INS_ vs. Teffs SPEC_INS_  Tregs UNSPEC_INS_ vs. Teffs UNSPEC_INS_  Tregs SPEC_B:9-23_ vs. Teffs SPEC_B:9-23_  Tregs UNSPEC_B:9-23_ vs. Teffs UNSPEC_B:9-23_ | 0.0084  0.0058  <0.0001  <0.0001  0.0019  0.0026  0.0031 |
| TNFRSF18 | Tregs POLY vs. Tregs UNSPEC_INS_  Tregs POLY vs. Tregs UNSPEC_B:9-3_  Tregs POLY vs. Teffs POLY | 0.0123  0.0393  0.0229 |

**Table S2.** Values of statistical significance for histone H3 modifications. Correlations not presented in the table did not show statistical significance.

| **methylation** | **compared cell populations** | **P value** |
| --- | --- | --- |
| H3K4me1 | Tregs POLY vs. Tregs UNSPEC_INS_  Tregs SPEC_INS_ vs. Tregs UNSPEC_INS_ | 0.0348  <0.0001 |
| H3K4me2 | Tregs POLY vs. Tregs UNSPEC_INS_ | 0.0427 |
| H2K4me3 | Tregs POLY vs. Tregs SPEC_INS_  Tregs POLY vs Tregs UNSPEC_B:9-23_  Tregs SPEC_INS_ vs. Tregs UNSPEC_INS_  Tregs SPEC_INS_ vs. Teffs SPEC_INS_  Tregs UNSPEC_INS_ vs. Tregs UNSPEC_B:9-23_ | 0.0417  0.0327  0.0120  0.0397  0.0328 |
| H3K9me1 | Tregs POLY vs Tregs UNSPEC_B:9-23_ | 0.0002 |
| H3K9me2 | Tregs SPEC_INS_ vs. Tregs UNSPEC_INS_  Tregs SPEC_INS_ vs. Teffs SPEC_INS_  Tregs UNSPEC_INS_ vs. Teffs UNSPEC_INS_ | 0.0431  0.0191  0.0141 |
| H3K27me1 | Tregs POLY vs. Tregs SPEC_INS_  Tregs SPEC_INS_ vs. Tregs UNSPEC_INS_  Tregs SPEC_INS_ vs. Tregs SPEC_B:9-23_  Tregs SPEC_INS_ vs. Teffs SPEC_INS_ | 0.0054  0.0141  0.0151  0.0215 |
| H3K27me2 | Tregs POLY vs Tregs UNSPEC_B:9-23_ | 0.0413 |
| H3K27me3 | Tregs POLY vs. Tregs SPEC_INS_  Tregs POLY vs Tregs UNSPEC_B:9-23_  Tregs SPEC_INS_ vs. Tregs UNSPEC_INS_  Tregs UNSPEC_INS_ vs. Tregs UNSPEC_B:9-23_  Tregs UNSPEC_INS_ vs. Teffs UNSPEC_INS_  Tregs UNSPEC_B:9-23_ vs. Teffs UNSPEC_B:9-23_ | 0.0385  0.0255  0.0163  0.0140  0.0267  0.0293 |
| H3K36me1 | Tregs POLY vs Tregs SPEC_B:9-23_  Tregs POLY vs Tregs UNSPEC_B:9-23_  Tregs SPEC_INS_ vs. Tregs SPEC_B:9-23_  Tregs SPEC_B:9-23_ vs. Teffs SPEC_B:9-23_  Tregs UNSPEC_B:9-23_ vs. Teffs UNSPEC_B:9-23_ | 0.0170  0.0461  0.0144  0.0050  0.0150 |
| H3K79me1 | Tregs POLY vs. Tregs SPEC_INS_  Tregs POLY vs Tregs SPEC_B:9-23_  Tregs POLY vs Tregs UNSPEC_B:9-23_  Tregs SPEC_INS_ vs. Tregs UNSPEC_INS_  Tregs SPEC_INS_ vs. Teffs SPEC_INS_  Tregs UNSPEC_INS_ vs. Tregs UNSPEC_B:9-23_  Tregs UNSPEC_INS_ vs. Teffs UNSPEC_INS_  Tregs UNSPEC_B:9-23_ vs. Teffs UNSPEC_B:9-23_ | 0.0445  0.0499  0.0069  0.0143  0.0088  0.0211  0.0116  0.0211 |
| H3K79me3 | Tregs POLY vs. Teffs POLY  Tregs SPEC_INS_ vs. Tregs UNSPEC_INS_  Tregs SPEC_INS_ vs. Teffs SPEC_INS_  Tregs UNSPEC_INS_ vs. Tregs UNSPEC_B:9-23_  Tregs UNSPEC_INS_ vs. Teffs UNSPEC_INS_ | 0.0343  0.0090  0.0389  0.0117  0.0406 |
| **acetylation** |  |  |
| H3K9ac | Tregs POLY vs Tregs UNSPEC_B:9-23_  Tregs UNSPEC_INS_ vs. Tregs UNSPEC_B:9-23_  Tregs UNSPEC_B:9-23_ vs. Teffs UNSPEC_B:9-23_ | 0.0486  0.0364  0.0101 |
| H3K14ac | Tregs SPEC_INS_ vs. Tregs SPEC_B:9-23_  Tregs SPEC_INS_ vs. Teffs SPEC_INS_  Tregs UNSPEC_INS_ vs. Tregs UNSPEC_B:9-23_  Tregs UNSPEC_INS_ vs. Teffs UNSPEC_INS_  Tregs UNSPEC_B:9-23_ vs. Teffs UNSPEC_B:9-23_ | 0.0152  0.0091  0.0441  0.0391  0.0342 |
| **phosphorylation** |  |  |
| H3ser10ph | Tregs UNSPEC_INS_ vs. Tregs UNSPEC_B:9-23_ | 0.0155 |
| H3ser28ph | Tregs POLY vs Tregs UNSPEC_B:9-23_  Tregs SPEC_INS_ vs. Tregs UNSPEC_INS_  Tregs UNSPEC_INS_ vs. Tregs UNSPEC_B:9-23_  Tregs UNSPEC_B:9-23_ vs. Teffs UNSPEC_B:9-23_ | 0.0406  0.0197  0.0102  0.0124 |
